# Effects of stachyose on absorption and transportation of tea catechins in mice: possible role of Phase II metabolic enzymes and efflux transporters inhibition by stachyose

**DOI:** 10.3402/fnr.v60.32783

**Published:** 2016-10-24

**Authors:** Wenfeng Li, Yalong Lu, Di Huang, Xiao Han, Xingbin Yang

**Affiliations:** Shaanxi Engineering Laboratory for Food Green Processing and Safety Control, College of Food Engineering and Nutritional Science, Shaanxi Normal University, Xi'an, China

**Keywords:** absorption, efflux transporter, Phase II metabolism, stachyose, tea catechins

## Abstract

**Background:**

Nutritional and absorption-promoting properties of stachyose combined with tea catechins (TC) have been revealed. However, the mechanism involved in non-digestible oligosaccharides-mediated enhancement of flavonoid absorption has largely remained elusive.

**Methods:**

This study was designed to investigate the molecular mechanism of stachyose in enhancing absorption and transportation of TC in mice. Mice were orally pre-treated with stachyose (50, 250, and 500 mg/kg·bw) for 0–8 weeks, and 1 h before sacrifice, mice were treated with TC (250 mg/kg·bw).

**Results:**

Gas chromatography-mass spectrometry analysis showed that serum concentrations of epicatechin, epigallocatechin, epicatechin gallate, and epigallocatechin gallate were dose- and time-dependently elevated with stachyose pre-treatment in mice. Furthermore, pre-treatment with stachyose in mice reduced intestinal sulfotransferase and uridine diphosphate-glucuronosyltransferase levels by 3.3–43.2% and 23.9–30.4%, relative to control mice, respectively. Moreover, intestinal P-glycoprotein and multidrug resistance-associated protein-1 contents were decreased in mice by pre-administration of stachyose in dose- and time-dependent manner.

**Conclusions:**

This is the first time to demonstrate that suppression of Phase II metabolic enzymes and efflux transporters of TC in the intestine can play a major role in increasing absorption of TC by stachyose feeding.

It is well recognized that tea is one of the most widely consumed beverages, next to water, in the world (1, 2). In the past few decades, tea has attracted much attention for its various physiological functions including anticancer, anti-arteriosclerosis, and hepatoprotective effects (3). These beneficial effects are associated with tea catechins (TC) closely, which consist primarily of four components, epicatechin (EC), epigallocatechin (EGC), epicatechin gallate (ECG), and epigallocatechin gallate (EGCG) (2, 4). However, extensive studies about the metabolism of TC suggest that bioavailability of TC *in vivo* is extremely poor by oral administration (3, 5, 6).

Interestingly, recent reports showed that non-digestible carbohydrates could promote absorption of diet polyphenols. For example, α-1,6-glucosaccharide promoted absorption of quercetin glycoside, and difructose anhydride III increased bioavailability of αG-rutin, and fructooligosaccharide enhanced absorption of α-glucosyl-isoquercitrin, genistein and daidzein, and chito-oligosaccharide improved absorption of phenolic acids of *Flos Lonicerae* extract (7–12). In addition, our recent research also revealed that stachyose, a non-digestible saccharide rich in soybean (*Glycine max*), *Rehmannia glutinosa* Libosch and *Lycopus lucidus* Turcz, could increase absorption and hepatoprotective effects of tea polyphenols in high fructose-fed mice (13). However, we still have a poor understanding of the mechanism that stachyose enhances absorption of TC.

It is interesting to note that intestinal and/or hepatic first-pass extraction via glucuronidation and sulphation, which are part of Phase II metabolism, can limit the oral bioavailability of flavonoids (14, 15). However, the activities of Phase II metabolic enzymes in the liver of rat were mediated by chitosan oligosaccharides and sulfated polysaccharide of *Monostroma nitidum*, suggesting that these non-digestible saccharides influenced Phase II detoxifying reactions in the liver (16, 17). In addition, the transport of ingested flavonoids across the intestinal barrier is also an important factor determining bioavailability upon oral intake (18). Previous reports suggest that the absorption extent of EC, ECG, and EGCG may be increased by inhibiting membrane transporters, mainly efflux transporters including P-glycoprotein (P-gp) and multi-drug resistance proteins (19–21). Interestingly, some non-digestible saccharides, such as hyaluronan oligosaccharides and astragalus polysaccharides, have recently been shown to down-modulate P-gp and multidrug resistance-associated protein-1 (MRP1) expression, thereby increasing the intracellular concentration of chemotherapeutic drugs (22, 23). In this regard, stachyose-elevated absorption of TC is likely associated with inhibition of first-pass metabolism of TC *in vivo*.

Therefore, the aim of the present study was to provide evidence that the feeding of stachyose actually inhibited the expression of efflux transporters and Phase II enzymes in the intestine and/or liver, and these suppressions contributed to the promotion of TC absorption in mice. In addition, the characterization that stachyose-enhanced absorption of TC was also investigated by evaluation of serum TC concentrations.

## Materials and methods

### Materials and reagents

Stachyose (pure>80%) was prepared in our laboratory (13). TC (pure>70%), which consist primarily of 3.11% EC, 4.05% EGC, 11.74% ECG, and 52.24% EGCG, were purchased from Yongqi Biology Co. Ltd (Xi'an, China) (13). Pure standards of EC (98%), EGC (98%), ECG (98%), EGCG (98%), and genistein (IS, 98%) were obtained from Sigma-Aldrich (Steinheim, Germany). Enzyme-linked immuno sorbent assay (ELISA) kit of P-gp was purchased from R&D Systems, Inc. (Shanghai, China). ELISA kits of the MRP1, MRP2, uridine diphosphate-glucuronosyltransferase (UGT), and sulfotransferase (SULT) were purchased from Shanghai enzyme-linked biological technology Co., Ltd. (Shanghai, China). ELISA kit of cytochrome P450 enzymes (CYPs) was obtained from Jiancheng Bioengineering Institute (Nanjing, China). N,O-bis(trimethylsilyl)-trifluoroacetamide (BSTFA) was purchased from Adamas Reagent, Ltd. (Shanghai, China). β-Glucuronidase (G0251-100KU, EC 3.2.1.31) and sulfatase (S9626-10KU, EC 3.1.6.1) were purchased from Sigma-Aldrich (Shanghai, China). All other chemicals were of the highest grade available.

### Animals

Male Kunming mice (15–20 g) and standard rodent chow were purchased from the Experimental Animal Center of the Fourth Military Medical University, China. They were housed in a controlled laboratory environment (22±2°C, relative humidity of 60±5%, and 12/12 h light–dark cycle). After 1-week acclimatization, the mice were divided randomly into seven groups with eight mice each (Table 1). Group I (normal group): mice were administered only intragastrically (i.g.) with physiological saline once daily during the experimental period for 8 weeks, and the serum EC, EGC, ECG, and EGCG were not detected in the normal mice. Control group II (Con. group): mice were treated with saline once daily for 8 weeks and were also administrated with TC at 250 mg/kg·bw (i.g., 0.4 mL) onetime 1 h before sacrifice.

**Table 1:** Experimental design

	Groups						
	
Administration	I	II	III	IV	V	VI	VII
Time for pre-treatment of stachyose (week)	–	–	5th~8th	5th~8th	5th~8th	7th~8th	1th~8th
Duration for pre-treatment of stachyose	–	–	4 weeks	4 weeks	4 weeks	2 weeks	8 weeks
Time for treatment of saline (week)	1th~8th	1th~8th	1th~4th	1th~4th	1th~4th	1th~6th	–
Stachyose (mg/kg·bw)^a^	–	–	50	250	500	250	250
TC (mg/kg·bw)^b^	–	250	250	250	250	250	250

a The treatment of stachyose at the last time was combined together with the administration of TC;

b The administration of TC was performed for mice 1 h before sacrifice. In addition, the TC was dissolved in water together with stachyose at relevant doses.

Groups III, IV, and V (dose-dependent test groups): mice were pre-treated with saline once daily for a continuous 4-week period and subsequently pre-treated with stachyose at 50, 250, and 500 mg/kg·bw (i.g., 0.4 mL) once daily for another 4 weeks, respectively; and finally, the mice of Groups III–V were also administrated with TC at 250 mg/kg·bw one time 1 h before sacrifice. To evaluate time-dependent effects of stachyose pre-treatment on absorption of TC, the mice in Groups VI and VII were administrated with stachyose at 250 mg/kg·bw once daily for 2 and 8 weeks, which began from the 7th and 1st week, respectively, and before the treatment of stachyose, the mice in Groups VI and VII were administrated with saline once daily. Meanwhile, the mice of Groups VI and VII were also administrated with 250 mg/kg·bw TC one time 1 h before sacrifice. During the administration period, all the mice were allowed free access to food and tap water. The animals were fully anesthetized by the inhalation of isoflurane and sacrificed to obtain plasma, liver, and small intestine samples. All the experiments were conducted according to the Guidelines of Experimental Animal Administration published by the State Committee of Science and Technology of People's Republic of China. The experiment was approved by the Committee on Care and Use of Laboratory Animals of the University (XJYYLL-2015689), China.

### Determination of serum catechins

Collected blood was centrifuged for 15 min at 1,500×g to obtain serum. The serum EC, EGC, ECG, and EGCG concentrations were evaluated by a gas chromatography-mass spectrometry (GC-MS) analysis method, which was described in our previous work with a minor revision (13). A 100 µL serum was incubated with 5 µL internal standard (genistein, 38.33 µg/mL), 5 µL PBA (200 mg/mL vitamin C and 62.5 mg/mL NaH_2_PO_4_), 50 µL 66.59 mg/mL CaCl_2_, 25 µL 20 UI/100 µL sulfatase, and 50 µL 200 UI/100 µL β-glucuronidase at 37°C for 90 min. After incubation, the serum was extracted twice with ethyl acetate (1.0 mL each time). The combined ethyl acetate extract was dried at 50°C for 50 min by a vacuum dryer (Rikakikai Co., Ltd, Tokyo, Japan), and then, the dried residue was redissolved in 60 µL pyridine and derivatized with 90 µL BSTFA at 70°C for 2 h.

One microliter derivatized extract was analyzed by a GC-MS 2010ultra (Shimadzu China Co., LTD., Shanghai, China) equipped with Rxi-5Sil MS Cap. column (30 m×0.25 mm id, 0.25 µm film thickness; Risetech Technology Co., LTD, Beijing, China). The flow velocity of carrier gas (helium) is 1.0 mL/min. The initial column temperature was 70°C for 4 min, and then ramped to 100°C at 4°C/min where it was held for 3 min, and increased to 260°C at 30°C/min where it was held for 20 min and further increased at 10°C/min to 295°C where it remained for 30 min.

### ELISA analysis

Intestinal tissue (0.5 g) or liver tissue (0.5 g) was homogenized with 4.5 mL saline and then centrifuged at 3,000 g for 10 min. In current work, the levels of P-gp, MRP1, MRP2, UGT, and SULT in intestinal tissue, and the levels of CYPs, UGT, and SULT in liver tissue were detected by commercial ELISA kits following the relevant manufacturer's instruction.

### Statistical analysis

Analytical results of serum catechins were presented as the means±SD. Statistical significance of difference between groups was performed by ANOVA followed by least significant difference test (LSD), which was performed by SPSS (20.0, IBM). *p<*0.05 was considered to be statistically significant.

## Results

### Stachyose promotes intestinal absorption of TC in mice

**Fig. 1:**
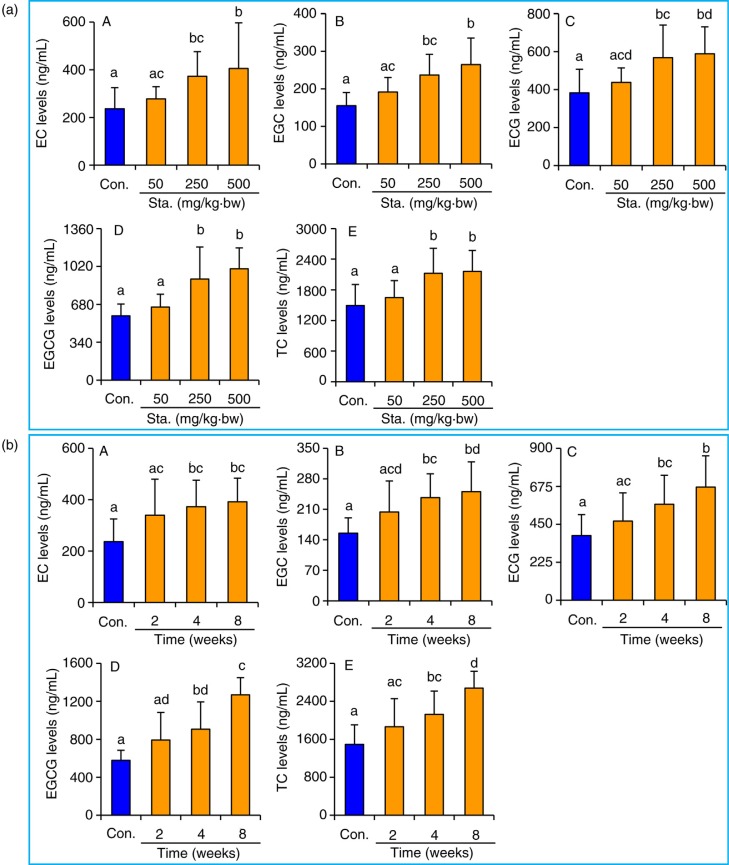
Pre-treatment of stachyose at 50, 250, and 500 mg/kg·bw for 4 weeks (a) increased serum concentrations of epicatechin (EC, a-A), epigallocatechin (EGC, a-B), epicatechin gallate (ECG, a-C), epigallocatechin gallate (EGCG, a-D), and total tea catechins (TC, a-E) in mice, respectively. Pre-administration of stachyose at 250 mg/kg·bw for different time (b) elevated serum contents of EC (b–A), EGC (b–B), ECG (b-C), EGCG (b-D), and total TC (b-E) in mice. Each value is expressed as the means±SD (*n*=8). ^a–d^Mean values with different letters are significantly different from one another (*p <* 0.05). Sta., stachyose.

To evaluate the effect of stachyose pre-treatment with different doses on absorption of TC, the mice were pre-treated with stachyose at 0, 50, 250, and 500 mg/kg·bw for 4 weeks and were subsequently administrated with TC at 250 mg/kg·bw 1 h before sacrifice. The serum levels of total TC and its monomer EC, EGC, ECG, and EGCG in the tested mice were determined by GC-MS, and the result is presented in Fig. 1a. Interestingly, the serum EC level was significantly increased by 57.5 and 71.3% in mice pre-treated with stachyose at 250 and 500 mg/kg·bw for 4 weeks, when compared with the control mice, respectively (Fig. 1a–A). Similarly, the serum EGC concentration was significantly elevated (*p<*0.05) in mice by pre-administration of stachyose at 250 and 500 mg/kg·bw for 4 weeks, and this effect can be performed in a dose-dependent manner (Fig. 1a-B). The pre-treatment of stachyose at a low dose of 50 mg/kg·bw led to a slight increase in the level of serum EC and EGC, but statistical difference was not significant (*p>*0.05). Furthermore, the pre-treatment with 250 and 500 mg/kg·bw stachyose in mice for 4 consecutive weeks notably elevated (*p<*0.05) serum ECG levels by 48.4 and 53.8%, and serum EGCG contents by 56.9 and 73.0%, respectively, and the effect was dose-dependent (Fig. 1a-C, a-D). Although pre-administration of stachyose at 50 mg/kg·bw led to slight elevation in serum ECG and EGCG levels as compared to the control mice, there was no statistical significance (*p>*0.05). As expected, pre-treatment of stachyose at 50, 250, and 500 mg/kg·bw for 4 weeks also dose-dependently elevated the total serum total TC level in mice (Fig. 1a-E).

To further test the time-dependent effect of stachyose ingestion on absorption of TC, mice were pre-treated with stachyose at 250 mg/kg·bw for 2, 4, and 8 weeks, and the mice were also orally administrated a single dose of TC (250 mg/kg·bw) 1 h before sacrifice. As can be seen in Fig. 1b-A and b-B, the serum EC and EGC levels in mice pre-treated with stachyose at 250 mg/kg·bw for 4 and 8 weeks were significantly higher (*p<*0.05) than in control mice. In addition, serum EGC concentration was also significantly (*p<*0.05) increased by 48.4 and 75.0% in mice pre-administrated with stachyose (250 mg/kg·bw) for 4 and 8 weeks, when compared to the control mice, respectively (Fig. 1b-C). It was noted that the EGCG concentration in the mice pre-treated with stachyose (250 mg/kg·bw) for 4 and 8 weeks was significantly (*p<*0.05) augmented to 906.06 and 1267.64 ng/mL from 577.59 ng/mL of control mice, respectively (Fig. 1b-D). Furthermore, the serum total TC content was also elevated with the increase of pre-treatment time of stachyose in mice (Fig. 1b-E). Although the serum EC, EGC, ECG, EGCG, and total TC contents in mice pre-treated with stachyose at 250 mg/kg·bw for 2 weeks were higher than that in control mice, it did not show statistical difference (*p>*0.05). It was also observed that single administration of stachyose (250 mg/kg·bw) together with TC (250 mg/kg·bw) 1 h before sacrifice did not significantly elevate (*p>*0.05) the serum catechins contents in mice, relative to control mice (data not shown). These results suggested that stachyose increased intestinal absorption of TC in a time-dependent manner.

### Effects of stachyose on intestinal Phase II enzymes

**Fig. 2:**
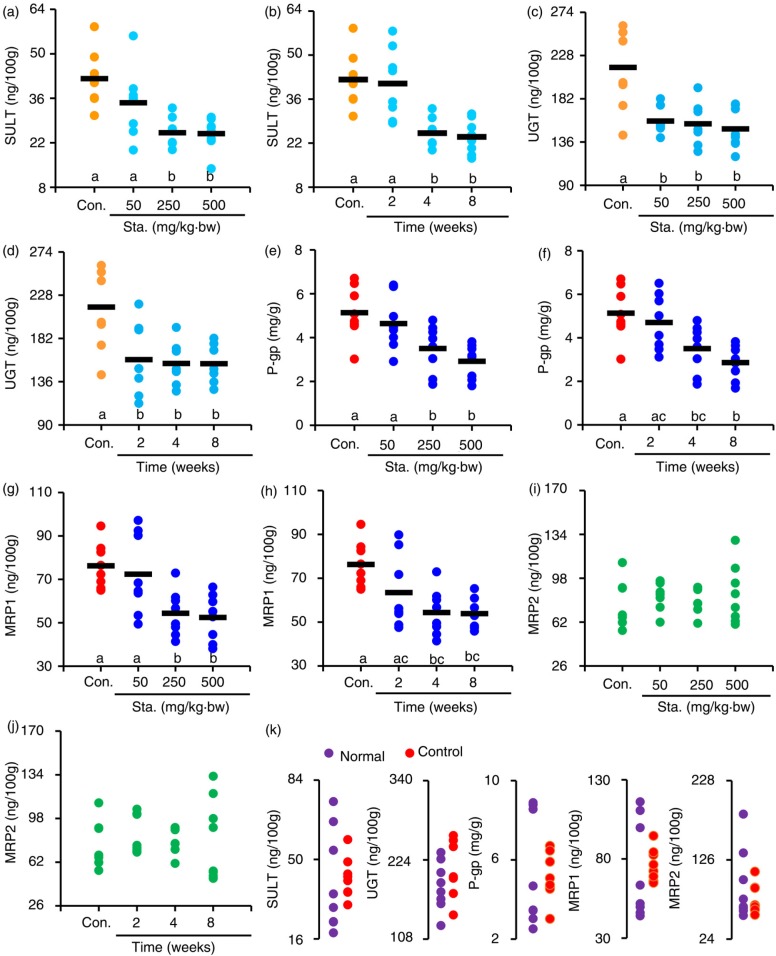
Effects of pre-treatment of stachyose at different doses for 4 weeks on intestinal SULT (a), UGT (c), P-gp (e), MRP1 (g), and MRP2 (i) levels, respectively. The effects of pre-treatment of stachyose at 250 mg/kg·bw for different persistent periods on intestinal SULT (b), UGT (d), P-gp (f), MRP1 (h), and MRP2 (j) levels, respectively. Intestinal SULT, UGT, P-gp, MRP1, and MRP2 levels were also compared between untreated normal mice and TC alone-treated control mice (k). Each point in a group represents a value from a sample, and its average value was represented as a horizontal line. The letters (a–c), which are displayed on the abscissa, denote that the mean values with different letters are significantly different from one another (*p <* 0.05).

In the enterocytes, the Phase II enzymes SULT and UGT are significant metabolic pathways for numerous endo- and xenobiotics and are also known to interact with the ingested flavonoids, which may accelerate efflux transportation of flavonoids, and thereby reduce bioavailability of flavonoids (15, 24). As shown in Fig. 2a, the SULT levels of the mice pre-treated with stachyose at 250 and 500 mg/kg·bw for 4 weeks showed a dramatic (*p<*0.05) decrease to 25.16 and 24.88 ng/100 g from 42.21 ng/100 g of control mice. Although the pre-treatment of stachyose at 250 mg/kg·bw for 2 weeks led to a slight decrease in the enteric SULT level (*p>*0.05), administration of stachyose (250 mg/kg·bw) for 4 and 8 weeks significantly decreased intestinal SULT levels (*p<*0.05) as compared to control mice (Fig. 2b). These findings suggest that the pre-treatment of stachyose can dose- and time-dependently inhibit intestinal SULT expression in mice. Besides, the intestinal UGT levels in mice were also analyzed by ELISA, and the results showed that the intestinal UGT level of mice pre-administrated with stachyose at all scheme was significantly lower than that of control mice (*p<*0.05, Fig. 2c, d).

### Stachyose inhibits expression of efflux transporters in mouse intestine

*Flavonoids* are known to interact with a number *of* membrane transporters, influencing *the* rate *of* intestinal nutrient *absorption, and* P-gp is a well-known efflux pump responsible for multiple drug resistance (25), and it plays a major role in the cells to transport catechins from intra-cell to outside (3). As depicted in Fig. 2e, the pre-administrations of stachyose at 50, 250, and 500 mg/kg·bw for 4 weeks and subsequent treatment of TC at 250 mg/kg·bw before sacrifice caused the dose-dependent decrease in P-gp expression of mouse intestinal tissue, showing the decrease by 9.6% (*p>*0.05), 31.7% (*p<*0.05), and 43.1% (*p<*0.05), relative to control mice, respectively. In addition, the P-gp expression in intestinal tissue of mice pre-treated with stachyose at 250 mg/kg·bw for 2, 4, and 8 weeks was also reduced to 523.01 (*p>*0.05), 389.71 (*p<*0.05), and 317.99 µg/g (*p<*0.05) from 570.82 µg/g of control mice, respectively (Fig. 2f).

MRP1 and MRP2 are also important membrane efflux transporters for Phase II metabolites of catechins (18, 26). As represented in Fig. 2g, the MRP1 levels in enterocytes of the mice pre-treated with stachyose at 50, 250, and 500 mg/kg·bw for 4 weeks were decreased to 72.43, 54.38, and 52.46 ng/100 g from 76.28 ng/100 g of control mice, respectively (*p>*0.05, *p<*0.05, *p<*0.05). We also observed a time-dependent decrease in intestinal MRP1 levels in mice pre-treated with stachyose at 250 mg/kg·bw for 4 and 8 weeks, when compared to the control mice (*p<*0.05, Fig. 2h). However, there were no prominent differences in the MRP2 levels in intestinal tissue among all mice (*p>*0.05) (Fig. 2i, j). It was worth noting that the individual administration of TC did not significantly affect the expression of intestinal SULT, UGT, P-gp, MRP1, and MRP2 in control mice (*p>*0.05 vs. the normal mice, Fig. 2k), indicating that the decrease of intestinal SULT, UGT, P-gp, and MRP1 levels in mice was not concerned with the administration of TC, and it was linked to pre-treatment of stachyose.

### Effects of stachyose on hepatic Phase II enzymes and CYPs

**Fig. 3:**
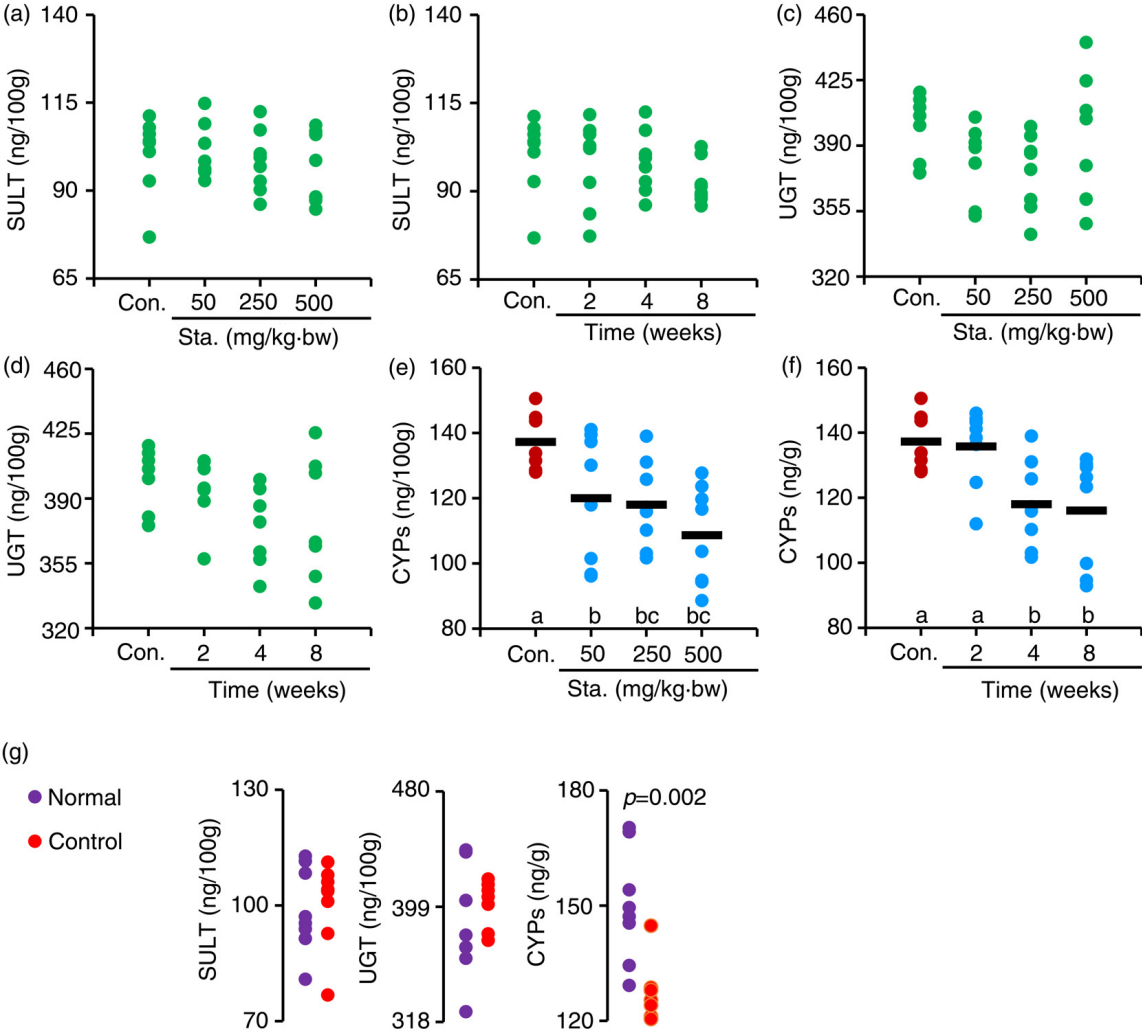
Pre-treatment of stachyose at different doses for 4 weeks on hepatic SULT (a), UGT (c), and CYPs (e) levels in mice, respectively. Pre-treatment of stachyose at 250 mg/kg·bw for different persistent periods on hepatic SULT (b), UGT (d), and CYPs (f) levels, respectively. Liver SULT, UGT, and CYPs levels in normal mice and control mice were also compared (G). The letters (a–c), which are displayed on the abscissa, denote that the mean values with different letters are significantly different from one another (*p <* 0.05).

The liver is an important organ for Phase I metabolism which is mainly catalyzed by CYPs, and also a major target for Phase II metabolism (glucuronidation and sulfation) of flavonoids (14, 24). Unexpectedly, our analysis showed that the hepatic SULT or UGT expression among all the tested mice showed no significant difference (*p>*0.05) (Fig. 3a–d). However, pre-treatment of 50, 250, and 500 mg/kg·bw stachyose for 4 weeks and subsequent treatment with 250 mg/kg·bw TC once before sacrifice of mice dose-dependently decreased the CYPs contents by 12.6, 14.0, and 20.8%, relative to control mice, respectively (Fig. 3e). In addition, the hepatic CYPs levels in mice pre-treated with stachyose at 250 mg/kg·bw for 2, 4, and 8 weeks were time-dependently reduced (*p<*0.05) by 1.1–15.4% as compared to control mice, respectively (Fig. 3f). Moreover, a dramatic decrease (*p<*0.05) was also seen in the expression of liver CYPs following the individual administration of TC in control mice, relative to normal mice (Fig. 3g). The results obtained in this analysis clearly indicate that liver CYPs levels are decreased by the administration of TC, suggesting that non-digestible stachyose might inhibit liver CYPs via improving the absorption of TC.

### Correlation analysis

**Fig. 4:**
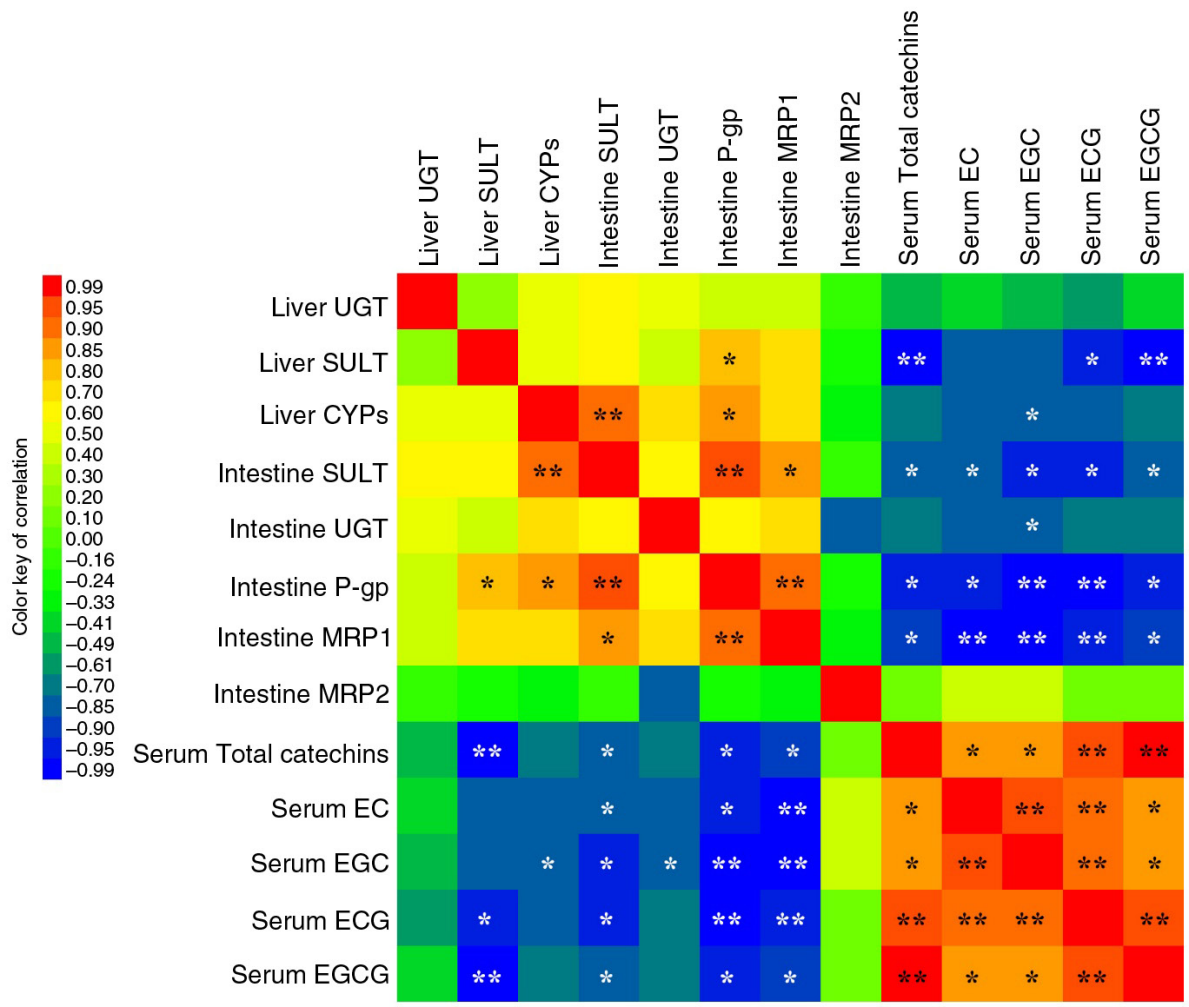
A pairwise correlations analysis was performed to integrate intestinal and hepatic Phase II metabolic enzymes and transport proteins of TC expression values with relative serum TC concentration in mice. The heat-map represents the correlation structure of dataset, which is highlighted with red (positive correlations) or blue (negative correlations) according to the color key on the right. * and ** indicate statistically significant correlation at *p*<0.05 and *p*<0.01, respectively.

To further understand the internal relation between serum catechin contents and the level of TC metabolism-related proteins, the identified data were assayed using correlation analysis, and the results are shown in Fig. 4. As expected, heat-map directly showed that negative correlations appeared between serum catechin contents and the level of TC metabolism-related proteins (SULT, UGT, P-gp and MRP1 in intestine, and SULT and CYPs in liver). This finding means that serum catechin concentrations increased with the decrease of intestinal SULT, UGT, P-gp and MRP1 levels, and hepatic SULT and CYPs levels in mice pre-treated with stachyose. It was also noticed that the levels of intestinal SULT, P-gp, and MRP1 were (*p<*0.05) negatively correlated with serum total catechins, EC, EGC, ECG, and EGCG contents, respectively. In addition, one significant negative correlation (*p<*0.05) was also presented between intestinal UGT level and serum EGC content, while the statistical significance was not found between intestinal UGT level and serum total catechins, EC, ECG, or EGCG concentrations, respectively. Moreover, liver SULT level was negatively (*p<*0.05) correlated with serum total catechins, ECC and EGCG contents, respectively. Although the hepatic CYPs levels decreased with an increase of serum catechin contents, there was only one significant correlation appeared between hepatic CYPs levels and serum EGC level. According to the correlation matrix, the intimate connection existed between elevation of serum catechin contents and suppression of expression of TC metabolism-related proteins in stachyose-treated mice.

## Discussion

Despite the abundant data revealing wide biological activities of dietary flavonoids, most flavonoids are poorly absorbed from the intestine and highly metabolized (27). Interestingly, previous research has suggested that non-digestible oligosaccharides can enhance intestinal absorption of polyphenols, which is possibly due to the fact that non-digestible oligosaccharides increase water-solubility of flavonoids and inhibit their degradation in gut (10, 28). It is also widely recognized that TC can undergo Phase II biotransformation mainly including UGT and SULT in intestinal epithelial cells, which may greatly contribute to their first-pass metabolism (14, 27, 29). In our hands, the mice pre-treated with stachyose dose- and time-dependently presented the reduction of intestinal SULT levels in mice (Fig. 2a, b). Additionally, all the administration strategies of the tested stachyose also caused the dramatic decrease in intestinal UGT levels in mice (Fig. 2c, d). The present results for the first time suggested that glucuronidation and sulfonation of TC were limited in intestinal tissue by pre-administration of stachyose, which might contribute to an increase in absorption of TC.

Intestinal membrane-bound efflux pump transports proteins, such as P-gp, MRP1, and MRP2, which play an important role in efflux transportation of Phase II metabolites of polyphenols, which is a main factor for first-pass metabolism of TC (18). It is also reported that absorption extent of catechins is closely connected with efflux pump transporters, suggesting that inhibition for the efflux transporters may contribute to bioavailability improvement of polyphenols upon oral uptake (18). MRP2 is an important efflux transporter, and inhibition of MRP2 may contribute to cellular uptake of EC, ECG, and EGCG (20, 21, 30). However, this work demonstrated that pre-administrations of stachyose could not reduce intestinal MRP2 levels in mice (Fig. 2i, j), suggesting that the enhancing of TC absorption was irrelevant to MRP2 in mice with feeding of TC with stachyose. However, it was suggested that the cellular uptake of EC, ECG, EGC, and EGCG could be increased by inhibition of P-gp and MRP1 (19–21). Herein, pre-treatment of stachyose was clearly shown to be the dose- and time-dependent decrease in intestinal P-gp and MRP1 expression (Fig. 2e–h), strongly suggesting that stachyose caused the inhibition for efflux transportation of TC in mouse intestine, which might contribute to enhancing the absorption of TC. Although TC was the potent inhibitor for Phase II metabolizing enzymes and efflux transporters (21, 29), this study suggested that the sole administration of TC did not affect the expression of SULT, UGT, P-gp, and MRP1 in mouse intestine (Fig. 2k), and the decrease of intestinal SULT, UGT, P-gp, and MRP1 levels in mice was possibly due to the pre-treatment of stachyose. As a result, this was the first investigation with unequivocal evidence that stachyose could inhibit the expression of intestinal SULT, UGT, P-gp, and MRP1 in mice, which is responsible for the enhancement of TC transportation in enterocyte, and thereby contributed to the elevation in bioavailability of TC.

In this study, we have provided robust evidence that supplementation of stachyose can significantly enhance absorption of TC, which is confirmed by GC-MS analysis, revealing that the serum EC, EGC, ECG, and EGCG, and total catechins contents are increased with the increases of dose and time of stachyose pre-treatment in mice (Fig. 1). Further, correlation analysis indicated that there was an important interplay between enhancing absorption of TC and expression inhibition of Phase II metabolic enzymes and efflux pump transporters in intestine by stachyose consumption (Fig. 4). This finding for the first time demonstrated that non-digestible stachyose-enhanced absorption of TC was closely associated with the inhibition of Phase II metabolism and efflux transport of TC by stachyose ingestion, and this might be a novel molecular mechanism for non-digestible saccharides-elevated bioavailability of diet flavonoids. It is widely understood that flavonoids, which are absorbed and transferred to the blood, can be further metabolized by hepatic SULT, UGT, and CYPs, and the metabolites will be excreted via the kidney swiftly, which may aggravate the decreased bioavailability (24, 31). Herein, the hepatic SULT and UGT levels were not different among all the mice in this study (Fig. 3a–d), suggesting that pre-treatment of stachyose did not change Phase II biotransformation of TC in the liver. Most drugs undergo an initial Phase I metabolism, generally catalyzed by CYPs, to form more water-soluble metabolites, and the metabolites are then catalyzed by UGT and SULT (32). However, previous studies suggest that catechins are not likely to undergo Phase I metabolism by CYPs due to their molecular structure (3, 32). Although catechins are not the substrate of CYPs, it is well known that TC can exhibit the strong inhibition of CYPs, and this effect may decrease related nutrients metabolism (32). In our study, the pre-treatment of stachyose dose- and time-dependently decreased hepatic CYPs levels in the mice (Fig. 3e, f). The present study also showed that the individual administration of TC decreased hepatic CYPs levels in mice (Fig. 3g), suggesting that TC could inhibit the liver CYPs expression. For this reason, the elevation of circulating catechins concentration caused by stachyose pre-treatment was suggested to intensify the down-regulation in hepatic CYPs expression, which was confirmed by pairwise correlation analysis. It is well known that humans and monogastric animals do not possess the α-galactosidase enzyme to break down stachyose, and therefore, stachyose cannot be absorbed to enter blood circulation (13, 33). Interestingly, our finding in this work strongly indicated that a potential molecular mechanism of health-promoting property of non-digestible stachyose *in vivo* was due to the capacity of stachyose to increase absorption of flavonoids.

In summary, this is the first report to show that pre-treatment of stachyose time- and dose-dependently increases the absorption of TC in mice. It also demonstrates, for the first time, that the suppression of Phase II enzymes (SULT and UGT) and efflux transporters (P-gp and MRP1) expression in intestine has a key role for enhancing absorption of TC by stachyose ingestion. All these findings may supplement the mechanism that non-digestible oligosaccharide enhances bioavailability of dietary flavonoids, and provide a scientific basis for co-application of stachyose with TC for health effects.
